# Living with HIV and Getting Vaccinated: A Narrative Review

**DOI:** 10.3390/vaccines11050896

**Published:** 2023-04-25

**Authors:** Andrea De Vito, Agnese Colpani, Mattia Trunfio, Vito Fiore, Giulia Moi, Marco Fois, Nicola Leoni, Stefano Ruiu, Sergio Babudieri, Andrea Calcagno, Giordano Madeddu

**Affiliations:** 1 Unit of Infectious Diseases, Department of Medicine, Surgery, and Pharmacy, University of Sassari, 07100 Sassari, Italy; colpani.agnese@gmail.com (A.C.); giordano@uniss.it (G.M.); 2 Unit of Infectious Diseases, Department of Medical Sciences, University of Turin, 10149 Torino, Italy

**Keywords:** HIV, vaccination, preventable diseases, HBV, HPV, HAV, SARS-CoV-2, MPox

## Abstract

After 40 years of its appearance, human immunodeficiency virus (HIV) infection remains a leading public health challenge worldwide. Since the introduction of antiretroviral treatment (ART), HIV infection has become a chronic condition, and people living with HIV could have life expectancies close to those of the general population. People with HIV often have an increased risk of infection or experience more severe morbidity following exposure to vaccine-preventable diseases. Nowadays, several vaccines are available against bacteria and viruses. However, national and international vaccination guidelines for people with HIV are heterogeneous, and not every vaccine is included. For these reasons, we aimed to perform a narrative review about the vaccinations available for adults living with HIV, reporting the most updated studies performed for each vaccine among this population. We performed a comprehensive literature search through electronic databases (Pubmed—MEDLINE and Embase) and search engines (Google Scholar). We included English peer-reviewed publications (articles and reviews) on HIV and vaccination. Despite widespread use and guideline recommendations, few vaccine trials have been conducted in people with HIV. In addition, not all vaccines are recommended for people with HIV, especially for those with low CD4 cells count. Clinicians should carefully collect the history of vaccinations and patients’ acceptance and preferences and regularly check the presence of antibodies for vaccine-preventable pathogens.

## 1. Introduction

After 40 years of its appearance, human immunodeficiency virus (HIV) infection remains a leading public health challenge worldwide. Since the introduction of antiretroviral treatment (ART), HIV infection has become a chronic condition, and people living with HIV (PWH) could have life expectancies close to those of the general population [1,2]. This implies that PWH are becoming older, with an increase in the comorbidity burden that HIV specialists have to manage [3,4,5,6,7,8,9,10,11,12]. However, despite ART, many people do not have a complete CD4 recovery [13,14,15,16], and PWH with low CD4 cell count have an estimated life expectancy of 30 years lower than the general population [1]. 

People with HIV often have an increased risk of infection or experience more severe morbidity following exposure to vaccine-preventable diseases. Therefore, it is fundamental to prevent non-communicable and infective diseases [17,18]. Nowadays, several vaccines are available against bacteria and viruses. Different vaccinal technologies have been developed over the years (e.g., live-attenuated, whole inactivate vaccine, virus-like particles, polysaccharide, mRNA), with varying routes of administration (e.g., oral, subcutaneous, nasal) [19,20]. However, not all vaccines could be administered in PWH, particularly in those people with a low CD4 cells count. For example, the use of a trivalent live-attenuated vaccine against measles, mumps, and rubella is contraindicated in PWH with a CD4 cell count <200 cell/mm^3^ for the high risk of developing the disease [21,22,23]. Therefore, it is clear that focusing on the vaccination of PWH is mandatory, balancing the pros and cons of each available vaccine. 

National and international vaccination guidelines in PWH are heterogeneous, and not every vaccine is included [22,23,24,25,26,27] (Tables 1, 2, 5–7 and 9). For these reasons, we aimed to perform a narrative review about the vaccinations available for adults living with HIV, reporting the most updated studies performed for each vaccine among this population.

## 2. Vaccines

### 2.1. Hepatitis A Virus

Hepatitis A is an acute infectious disease transmitted through contaminated food or water by fecal–oral route. Hepatitis A virus (HAV) has an icosahedral structure, and human beings are the only reservoir in its biological cycle. Therefore, it is a cosmopolitan infection. However, its circulation is influenced by hygiene and socioeconomic condition; thus, it is an endemic disease in developing countries commonly acquired during childhood. In non-endemic countries such as Europe, HAV infection is acquired in adulthood. Risk factors include travel to an endemic country, intravenous drug use, or homelessness and being men who have sex with men (MSM) [28]. 

No specific disease manifestations in immunocompromised hosts or PWH have been described. However, Lin et al. showed that people with HIV are more infectious and for a more extended period than people without HIV [29]. Therefore, the normalization of transaminase levels may also be prolonged in these patients [30]. However, some cases of fibrosing cholestatic hepatitis in PWH with severe immunodeficiency have been described, with severe jaundice, coagulation deficit, and encephalopathy with rapid evolution until death [31].

Specific treatment for HAV is not available. Hospitalization is mandatory only in fulminant hepatic failure, requiring a liver transplant. Predictors of disease evolution are age (<10 or >40 years old), creatinine ≥2–3 mg/dl, and prothrombin time ≥50 s. Otherwise, supportive care and symptomatic treatment are sufficient [32].

Prevention measures for HAV infection include vaccination, immunoglobulin administration, and careful personal hygiene. Vaccination of high-risk adults such as travellers in super-endemic countries, MSM, patients with chronic liver disease, and individuals with one year or more of HIV infection is recommended. The HAV vaccine contains purified and inactivated viruses boosted by an aluminium salt as an adjuvant. Two doses are required in a range of six to eight months [33]. The vaccine is safe and immunogenic in 97–99% of the cases two weeks after the second dose. However, the efficacy is inferior in HIV co-infected patients, and the seroconversion rates range from 52% to 94% [34]. Recommendations of the different guidelines are summarized in Table 1.

Patients with CD4 cells count <200 cells/mm^3^ and those with detectable HIV-RNA have a higher risk of poor response to the vaccine. For these reasons, some authors suggest an additional dose of the HAV vaccine to improve the durability of seroprotection in PWH with low CD4 cells count [29]. Chen et al. found that PWH who have lost their anti-HAV antibodies after primary vaccination had a faster and better serological response to a single dose of HAV revaccination than PWH who received the first dose [35]. Regarding the durability of HAV response, Jablonowska and Kuydowicz evaluated 234 PWH, of which about 30% had anti-HAV antibodies [36]. Of the 83 PWH who received a complete vaccination, 79.5 had a good response (anti-HAV-T >20 IU/L after one month since the booster dose). In addition, they confirmed that having less than 200 CD4/mm^3^ and HIV/HCV coinfection were associated with a worse response [36].

The most common adverse events are fever, injection site reaction, rash, and headache [37]. In addition, severe events, such as Guillain-Barrè syndrome, have been reported, although their relationship with vaccination is uncertain. No difference regarding safety has been seen in PWH [34]. Finally, Crisinel et al. reported a 100% seroconversion rate after two doses of HAV vaccination in children living with HIV without severe symptoms or immunosuppression. Despite a high seroconversion rate, children with CD4 counts of <750/mm^3^ have lower anti-HAV antibodies, which could reflect less lasting protection. For this reason, serological monitoring and additional boosting doses should be considered for these children [38].

Although vaccination against HAV is essential for HIV-infected patients, the uptake of HAV vaccine is reported to be very low [39]. For this reason, further efforts are needed to improve HAV vaccine offer and acceptance. 

### 2.2. Hepatitis B Virus

Hepatitis B virus (HBV) is one of the principal causes of chronic viral liver disease worldwide. As for HIV, no definitive cure is currently known. 

Hepatitis B virus could be transmitted by blood, semen and other bodily fluids, or through vertical transmission [40,41,42,43]. People with HIV are less likely to develop a clinical recovery after acute infection, given the lower HBV surface antibody (HBsAb) production rates. For this reason, these patients are more likely to develop a chronic infection, with liver cirrhosis, end-stage liver disease, and hepatocellular carcinoma (HCC) than the general population [44,45,46,47,48,49]. 

HBV surface antibody production is less commonly observed with low CD4+ cell counts, high HIV viral loads, HCV co-infection, and other comorbidities [50]. 

Even in the ART era, there is evidence of higher mortality among PWH with HBV infection [51]. For this reason, achieving long-term immunity protection is still challenging, and vaccination remains one of the most important weapons for HBV prevention [52]. Consequently, targeted interventions for assessing immunity and primary prevention with vaccines should be prioritized, especially for those people who need to switch to a dual-regimen treatment without an active drug against HBV. 

Regarding vaccination, there available different effective formulations. They consist of recombinant, combined, or mammalian cell-derived vaccines.

*(i)*  Recombinant HBV vaccines include Recombivax HB^®^ [containing 10 mcg HBV surface antigen (HBsAg)/mL], Engerix-B^®^ (containing 20 mcg HBsAg/mL), and Heplisav-B^®^ (HepB-CpG; containing 20 mcg HBsAg/0.5 mL). Recombivax HB^®^ has been available since 1983 and is widely available [53]. It typically requires three doses. Engerix-B^®^ has been available since 1989 and usually requires three doses. Finally, HepB-CpG (an adjuvanted vaccine) was approved in 2017, only in adults. It is administrated in two separate doses at one month of distance [54,55,56].
*(ii)*  Combined vaccines: The combined vaccine (Twinrix^®^) contains 720 enzyme-linked immunosorbent assay units of inactivated hepatitis A virus and 20 mcg of recombinant HBsAg [57].

The different recommendations are summarized in Table 2.

According to all national and international guidelines, PWH should be assessed for HBV infection before choosing vaccination. In particular, every patient should be screened for HBsAg, HBsAb, and HBV core antigen–antibody (HBcAb) [23,24,25,26,58]. Depending on the results of this test, we could face different situations:

*People with HIV who never received HBV vaccine and who have never encountered the virus* (anti-HBs, HBsAg, anti-HBc negative)

In this case, HBV vaccination is recommended for all PWH regardless of HIV viral load (VL) and CD4+ cells count, given their HBV susceptibility. 

Four randomized controlled trials (RCTs) on PWH were conducted from 2005 to 2013, analyzing various schedules [59,60,61,62]. In almost all cases, there was an advantage to a higher dose of vaccine in this population. Interestingly, Cornejo-Juárez et al. showed no relevant differences when comparing a 10 µg to a 40 µg schedule [60]. Furthermore, an RCT conducted by Chaiklang et al. in 2013 highlighted no statistical differences between the study groups; they also observed a higher response rate in classic schedules than in every previous study [63]. However, results showed a response ranging from 16% to 18.2% lower at 12 months in the group receiving the standard dose (Table 3). Even if not statistically significant, this result could be considered clinically relevant when coming to practice. Finally, in 2013, a meta-analysis carried out by Ni et al. confirmed the advantage of higher doses to increase response rates (pooled OR for increased dose = 1.96 (95% CI 1.47–2.61)) [64].

*People with HIV who did not respond to a vaccine cycle* (anti-HBs, HBsAg, anti-HBc negative with a known history of vaccination)

People with HIV who did not respond to a vaccine cycle are considered susceptible to HBV. For this reason, a new vaccine cycle should be considered for this population to obtain an acceptable serological response. However, there are no univocal indications regarding revaccination or the schedule to use. For example, in the United States and in France, a second series is recommended [23,65]. Instead, British recommendations consider three vaccines with high doses [25].

Over the years, several non-randomized studies have been conducted, but only a few RCTs.

In 2010, Psevdos et al. analyzed the response differences between supplementary double or standard doses among 101 PWH non-responders to the first HBV vaccine schedule [66]. They found a difference of ~30% in response rates in favor of double dose (*p* = 0.006). 

In 2015, Rey et al. conducted a study on 178 PWH assessing the efficacy of a double vs. standard dose vaccine among non-responders to a 20 µg booster after the first vaccine cycle [67]. As a result, they found a significant difference at week 72 of follow-up (54% vs. 31%, respectively; *p* = 0.01). 

Recently, Vargas et al. compared the efficacy of a high-dose vs. standard-dose HBV revaccination schedule in an RCT including 107 PWH. From December 2013 to March 2018, they enrolled patients with HBs-Ab titers <10 IU/L after the first HBV vaccination regimen [68]. The high-dose group received three doses of 40μg recombinant HBV vaccine; the other one received 20 μg at 0, 1, and 2 months. At one year of follow-up, they demonstrated 40% higher response rates among people receiving a 40 μg dose (80% vs. 39.1%; *p* = 0.01) (Table 4).

All this considered, a double-dose revaccination seems to be the best practice among PWH who did not respond to the first vaccine cycle.

*People with HIV with positive HBcAb*

Literature data showed how up to 20% of PWH tested positive for HBcAb [69,70,71,72,73] However, there is no international consensus about vaccination in this case. For example, the European AIDS Clinical Society (EACS) guidelines report that “vaccination is not recommended in this population” [21], while the National Institute of Health (NIH) suggest performing one standard dose of HepB (Table 2). 

Unfortunately, few studies about this population are present. In 2003, Gahndi et al. found that among 42 PWH positive only for HBcAb, one had a positive HBV-DNA, supporting the idea that occult hepatitis B viremia may occur even after apparent clearance of infection [74]. In 2016, Piroth et al. conducted a clinical trial in this population, enrolling 54 PWH with isolated HBcAg positivity. All people received one dose (20 µg) of the recombinant HBV vaccine. At 4 weeks, only 25 (46%) patients were responders, and only 14/24 (58%) maintained an anti-Hbs level >10 mIU/mL at 28 weeks (one LTFU). Those who at 4 weeks were non-responders (anti-HBs level of <10 mIU/mL) received three additional double doses. Among them, 24/27 (89%) and 81% (21 of 26) had an anti-HBs level of ≥10 mIU/mL at week 28 and month 18, respectively. The authors concluded, “All of the patients with an isolated anti-HBc profile who did not have an anti-HBs titer of >100 mIU/mL 4 weeks after a single recall dose of HBV vaccine should be further vaccinated with a reinforced triple double-dose scheme” [75]. Finally, from 2005 to 2016, some prospective studies aimed to evaluate response rates with different schedules, and vaccine success was reported in up to 89%. For this reason, we agree with NIH guidelines since the HBV vaccine represents an added value among these patients, reducing the risk of new infections [74,75,76,77].

*HBV surface antigen positive*

In this case, vaccination is not recommended, and patients should be treated with a triple-drug regimen containing two Nucleoside Reverse Transcriptase Inhibitors (NRTI), as suggested by guidelines [23,24,25].

In conclusion, the management of HBV vaccination is still debated in the literature, and further studies with longer follow-ups are needed. In the meantime, the suggestion is to monitor the HBsAg title yearly and use a tiple-drug treatment containing tenofovir in the non-responder subjects.

### 2.3. Human Papillomavirus

Human papillomavirus (HPV) is the most common sexually transmitted disease worldwide. Among the >200 identified genotypes so far, most can cause anogenital warts and respiratory papillomatosis, whereas about 40 genotypes have been associated with premalignant and malignant lesions of the cervix, anus, vulva, vagina, penis, and oropharynx [78]. Worldwide, genotypes 16 and 18 cause about 70% of cervical cancers (the fourth most common cancer in women), while HPV-6 and 11 are the most frequent causes of benign lesions [78,79].

People with HIV, even on effective ART, have increased risk and rate of HPV acquisition, persistence, and re-infection after clearance, higher carriage of multiple HPV genotypes, and a more rapid progression to HPV-associated malignancies [80,81,82]. Furthermore, HIV-positive MSM have the highest HPV-related anal warts and cancer risk. At the same time, women with HIV show a six-fold greater incidence of cervical cancer than HIV-negative women [83]. The worse epidemiology of HPV infection in PWH is due to behavioral habits and immunological reasons related to HIV-induced NK, B, and T-cell dysfunction, chronic inflammation, and persistent mucosal/epithelial alterations [84]. Furthermore, HPV infection can increase by two-fold the likelihood of HIV acquisition [85]. HPV vaccination may have a relevant impact in settings featured by low HIV prevention coverage, where mathematical models showed that the cumulative number of HIV infections that could be averted by HPV vaccination over 50 years could reach up to 27,812 cases in women and 14,693 cases in men [86]. 

Four types of prophylactic recombinant vaccines based on virus-like particles are currently available: two bivalent (Cervarix^®^, Glaxosmith-Kline, UK, and Cecolin^®^, Xiamen Innovax Biotech, China), the quadrivalent, and the nonavalent (Gardasil^®^ and Gardasil-9^®^, Merck, Rahway, NJ, USA). The two bivalent vaccines protect against HPV-16 and 18, while Gardasil^®^ and Gardasil-9^®^ protect against HPV-6, 11, 16, and 18, and against HPV-6, 11, 16, 18, 31, 33, 45, 52, and 58, respectively. 

In the general population, HPV vaccines showed outstanding safety and effectiveness against vaccine-included genotypes, anogenital warts, and high-grade intraepithelial neoplasia up to 14 years after vaccination [87]. As a result, vaccination regimes for young girls and boys (9–14 years) have moved from the original licensed 3 doses to 2 doses. In addition, there is growing interest in evaluating whether one shot is insufficient for long-lasting protection [88].

Despite HPV vaccination being also recommended for immunocompromised subjects, including PWH, robust consolidated evidence on its efficacy and safety is missing in PWH. Several studies suggest these vaccines are safe and immunogenic. Still, there has been no formal assessment of the influence of vaccine type, number of doses, baseline HPV serostatus, nor of the age and timing of vaccination along the course of HIV infection stages. Guideline recommendations are summarized in Table 5.

A 2022 meta-analysis of 18 longitudinal studies, including about 3900 participants, evaluated HPV vaccines immunogenicity, safety, and efficacy in PWH according to baseline HPV status [89]. Overall, their findings support that PWH develop a valid immune response following HPV vaccination and that all the vaccines are as well tolerated and safe as for HIV-negative populations. Nevertheless, the pooled follow-up was 1–2 years, no study assessed the effects of one or two doses only, and only one reported on Gardasil-9^®^ and none on Cecolin^®^, recently licensed [89]. Furthermore, most of the participants included in the meta-analysis were relatively healthy but also more representative of old HIV-positive cohorts compared to PWH attending clinics nowadays: they presented an average long duration of infection off ART and old ART regimens.

As for immunogenicity, among PWH who were seronegative for HPV-16 and -18 prior to vaccination, seroconversion rates were high (>94%) at 7 months from the first dose across all vaccines [89]. Seropositivity after the third dose remained high despite some decline over time, which was more pronounced in PWH as compared to HIV-negative participants and greater for HPV-18 and with the quadrivalent vaccine; consequently, increases in seropositivity after a fourth dose was more pronounced for this genotype and with Gardasil^®^ [89]. Due to heterogeneity and limited statistical power, there was only modest evidence suggesting that antibody titers and seroconversion rates were lower in PWH with lower CD4+ counts or detectable plasma viremia. Therefore, no conclusion was drawn for the potential contributions of ART [89]. Previously, lower antibody titers and seroconversion rates after HPV vaccination in PWH with CD4+ count ≤200 cells/mm^3^, positive correlations between CD4+ count and antibody titers, no difference in antibody titers by CD4 nadir, and higher antibody titers in PWH on ART (vs. off ART) and in those virally suppressed (vs. non-suppressed) were reported [90,91,92,93,94]. Interestingly, genotype- and timing-specific differences in seropositivity between HIV-negative and PWH after three doses could also occur [89].

To date, no reliable estimate of any biological effect of HPV vaccination in PWH has been carried out; thereby, most evidence relies on immunological responses with no robust and unbiased data about the clinical counterpart of such responses, such as post-vaccination cytology results or rates of HPV infection and anogenital warts and cancers [89]. For instance, and partially differing from the results in adult PWH, among perinatally infected youths receiving Gardasil^®^, viro-immunological parameters were marginally associated with abnormal cytology but not with antibody titers or vaccine doses [95]. Eventually, the 2022 meta-analysis concluded that PWH who have not been vaccinated prior to acquiring HIV can still benefit from receiving the vaccine. However, the higher likelihood of HPV-positivity in PWH may hinder the net benefit of vaccination independently from other determinants, such as immune competence [89].

More data are also required to confirm the alleged superior immunogenicity of Cervarix^®^ compared to the other HPV vaccines in PWH [81,88,96]; this observation could be potentially explained by the fact that among the licensed vaccines, only Cervarix^®^ contains AS04. This peculiar adjuvant is a detoxified form of lipopolysaccharide that acts as a Toll-like receptor 4 (TLR) agonist. Previous evidence about anti-HBV vaccination suggested that TLR agonist adjuvant-based vaccines can improve PWH immunogenicity by overcoming T follicular helper dysfunctions [81,97]. However, considering the greater prevalence of infections by concurrent multiple HPV genotypes in PWH, the cross-protection towards a wider spectrum of genotypes induced by Cervarix^®^ may be lower compared to quadri- and nonavalent vaccines. Therefore, mixed vaccination regimens may be regarded with interest in PWH. However, the general population still has limited data supporting this approach [98]. 

In conclusion, further research is warranted to detail the influence of prior HPV exposure, the role of immune suppression, HIV infection stages, the timing of ART initiation and its duration, and the age of vaccination, as all could potentially affect the efficacy and duration of protection in PWH.

### 2.4. Influenza Virus

Human Influenza A and B viruses cause annual outbreaks of Influenza in temperate climates during winter. Influenza C virus is less common and causes milder diseases, while Influenza D virus does not affect humans. Seasonal Influenza symptoms include dry cough, fever, myalgia, arthralgia, headache, and malaise [99]. Most people recover without medical attention; however, high-risk individuals can develop severe illnesses and death. Mortality is estimated to be between 290,000 and 560,000 deaths per year. People at greater risk of disease progression are pregnant women, children under 59 months of age, people with chronic morbidities, elderly people, and people affected by immunosuppressive conditions, including PWH. Transmission easily occurs through droplets and can be prevented with face masks and frequent hand hygiene [100].

A safe and constantly updated inactivated vaccine is available, and its administration is recommended to all people aged >6 years, with high priority among at-risk individuals. A trivalent vaccine containing two strains of virus A and one of virus B was first introduced; in 2013–2014, a fourth component targeting B strain was added. The vaccine is not always consistent with the circulating virus due to the high variability of Influenza virus. However, it is updated twice yearly. Even if not perfectly matching, it can still confer protection against severe illness and hospitalization [101]. 

People with HIV have always been at higher risk of disease progression, even if hospitalization due to Influenza has decreased since the introduction of ART. Still, PWH are considered at higher risk of disease complications and severe illness; thus, annual vaccination with a tetravalent vaccine is recommended [102,103,104]. 

A systematic review by Remschmidt et al. conducted in 2014 aimed to assess Influenza vaccine safety and efficacy among PWH. Overall, they collected two randomized clinical trials, three cohort studies including adults with HIV, and one trial including children. All data collected refer to the trivalent vaccine, the only one available at the research time. Authors highlighted that the vaccine prevents Influenza in adults, but no evidence regarding pneumonia, hospitalization, and mortality was reported. No difference according to CD4+ cells count and HIV viral load was encountered.

On the contrary, effectiveness among children under six years old was not demonstrated. This group reported an inferior antibody response compared to its healthy counterparts. However, few data are available among children with and without HIV [105]. 

A cohort study conducted during seasonal Influenza 2013–2014, 2014–2015, and 2015–2016 involving PWH and HIV-negative subjects grouped by age showed a lower prevalence of vaccine responders among PWH. No clinical outcome was evaluated [106]. An analysis from the same cohort shows that dysfunctional peripheral antigen-specific T helper cells are associated with this impaired response and may be affected by ageing and HIV infection [107].

Regarding the tetravalent inactivated vaccine, no data regarding PWH are available. However, encouraging data from the trivalent vaccine among PWH and data regarding the tetravalent vaccine among the general population support safety and efficacy of the tetravalent vaccine among PWH [108,109]. 

Regarding safety, no differences have been seen in the adverse events in PWH and the general population. On the impact of vaccination on HIV viral load and CD4 cell count, controversial results have been published. Some authors showed HIV viral load rebound and a decrease in CD4 cell count after vaccination for Influenza. The HIV rebound has been attributed to the activation of quiescent HIV-infected CD4 cells. However, most of these studies were conducted between 1995 and 2010, when Integrasis inhibitors were unavailable [110].

The available literature pushes towards promoting vaccination among PWH. However, further studies are needed to assess the safety and efficacy of the quadrivalent inactivated Influenza vaccine among children and adults living with HIV.

### 2.5. Measles Morbillivirus

Measles is a severe disease caused by a Morbillivirus. It is highly contagious and particularly threatening for immunocompromised individuals [111,112,113,114]. Its incidence has been decreasing since the introduction of an effective vaccine; however, the reluctance to vaccine uptake is undermining the achievement of herd immunity in many countries [115,116]. Measle vaccination is part of the routinely administered vaccines during childhood. Its immunogenicity and safety among children living with HIV and exposed uninfected children have been studied in past years. 

A systematic review published in 2019, including only randomized control trials and cohort studies with HIV-negative children matching cohort, reported good immunogenicity with greater waning in children with HIV [117]. This is consistent with what was reported for other vaccines given before immune recovery. Immunologic recovery, intended as the recovery in the CD4 cell count, in children, is achieved through naïve cells; thus, immunity acquired with vaccines administered before immunologic recovery cannot be re-established (differently from what we can see in adults PWH). These findings support the administration of a booster vaccine after immunologic recovery. 

Interesting data emerged from another review from the same period, including cross-sectional studies, case reports, and case series. The Authors reported pooled data suggesting a better response to the vaccine from children on ART and with rapid ART initiation. In comparison, a poorer response was recorded when ART was differed or not prescribed. Despite the small sample size and the numerous confounders, these data reinforce the need to start treatment promptly in children living with HIV [118]. In addition, confirmation came from a recently published prospective study; during a two-year follow-up, Bruzzese et al. reported an 87% coverage among children with HIV on stable ART, with better response among children who received the vaccine after starting ART [119]. Regarding safety, no serious adverse events were reported in the two reviews. However, long follow-up data on immunogenicity and long-term efficacy are missing [117,118]. 

Regarding adults and adolescents, Loevinsohn et al. published a systematic review in 2019, including 9607 PWH. Immunogenicity was highly variable across the studies, significantly improving after ART introduction. However, despite complete ART coverage, the waning of immunity reaching 50% was also reported. Nevertheless, no severe adverse events were reported [120]. In this regard, only a case of vaccine-strain severe pneumonia in an HIV-infected young adult is well known; in this case, the CD4+ cell count was “too few to enumerate” when receiving the vaccine [121]. 

Studies reported are widely variable, and the characteristics of people included are heterogeneous; thus, more rigorous investigations regarding immunogenicity, efficacy, and safety of the measles vaccine among adults PWH are advocated. Despite the lack of punctual data on long-term immunogenicity and efficacy, based on severe outcomes of Morbillivirus infection and safety and immunogenicity data from cohort studies and randomized trials, the measles vaccine is recommended by HIV guidelines in PWH with CD4+ count >200 cells/mm^3^ and on stable ART [102,104]. Given the low rate of Morbillivirus antibodies among the population [122,123,124], far from reaching herd immunity, the pros and cons of recommending vaccination should be considered when evaluating a new patient.

### 2.6. Mpox Virus

Mpox virus (MPV) is a zoonotic orthopoxvirus that is in the same genus as variola virus (causative agent of smallpox), vaccinia, ectromelia, camelpox, and cowpox viruses. In particular, MPV is considered the most important orthopoxvirus infecting human beings since the eradication of smallpox, confirmed by WHO in 1980 [125]. 

MPV was identified in 1958; however, the first documented human infection was described in 1970 in a 9-year-old child from the Democratic Republic of Congo [126]. 

Since 1970, monkeypox has continued spreading in central and western Africa, which is now endemic. The first outbreak in Western countries was in the United States in 2003. Since then, sporadic outbreaks have been reported in several countries [127]. The most recent outbreak dates to May 2022 and is still ongoing; in this case, however, the person-to-person transmission would seem to be the main route of contagion, unlike the previous outbreaks [128].

The disease caused by MPV infection is usually characterized by a febrile prodrome with lymphadenopathy, headache, and fatigue (typically 0 to 5 days) followed, after 1 to 3 days, by a vescicopapular rash with lesions that generally start on the face and then spread to the whole body (including palms and soles). In addition, many atypical cases have been reported during the current outbreak, with skin lesions mainly localized at oral and anogenital levels, probably due to the sexual transmission route [129]. 

In most cases, the infection progresses benign, with complete healing after 2–4 weeks. However, cases of severe disease and complications have been described. The risk of developing a more serious disease also correlates with the patient’s immune status.

From preliminary data regarding the current outbreak, it seems to be a connection between MPV infection and HIV. Thornhill et al. reported that of 528 people infected between April and June 2022, 41% had HIV infection [130]. 

Even before 2022, data from Africa had shown that in people with uncontrolled HIV, especially when they presented with AIDS features, the course of monkeypox was more severe (e.g., more extensive lesions, more significant complications, and increased mortality) [131]. On the contrary, this discrepancy has not been highlighted for PWH on ART [132]. 

Currently, there are two types of vaccines against smallpox and MPV: a replication-deficient modified vaccinia Ankara (MVA) vaccine and a replication-competent smallpox vaccine (ACAM2000). 

The MVA vaccine is a second-generation smallpox vaccine. It represents the first choice due to its excellent safety profile, even in immunocompromised people. It is administered subcutaneously in two doses, 28 to 35 days apart. However, intradermal administration might be considered in outbreak situations if supplies are limited, as it requires a lower dose [133].

Greenberg et al. evaluated the safety and immunogenicity of MVA as a smallpox vaccine with a phase I/II clinical study comparing the safety and immunogenicity of MVA in 91 vaccinia-naive HIV-infected subjects (CD4+ T-cell counts, >350/mm^3^) and 60 uninfected subjects [134]. To measure the potential efficacy of MVA, the ability to boost the memory response in people previously vaccinated against smallpox was evaluated by enrolling vaccinia-experienced HIV-infected and HIV-uninfected subjects in two additional groups [134]. They found that MVA was well tolerated and immunogenic in all subjects, with an antibody response comparable between people without HIV (PWoH) and PWH. In 2020, Overton et al. conducted a phase II trial on PWH, enrolling 87 participants [135]. They were divided into three groups: (i) people who received two standard doses on weeks 0 and 4; (ii) people who received two standard double doses on the same schedule in the double dose; (iii) people who received standard doses on weeks 0 and 4 weeks, plus a standard boosted dose at week 12. No differences in safety and immune response have been reported in the three groups. Therefore, the authors concluded that a booster dose does not appear necessary.

On the contrary, Pugliese et al. propose to perform the third dose for people with a CD4 cells count below 200 cells/mm^3^ or 15% [136].

Agunbiade et al. recently conducted a cohort study of 10,068 (including also PWH) high-risk people who received MVA-BN vaccination [137]. They registered only 15 cases of Mpox, and 3 were HIV-positive. To note, the median time between vaccination and Mpox occurrence was 4 days (IQR 3–9).

The ACAM2000 vaccine, approved in 2007 to replace the original Dryvax vaccine used to eradicate smallpox, is a replication-competent vaccine with high immunogenic power. However, it can only be used in specific cases due to the frequent occurrence of severe adverse events secondary to the injection (acute vaccinia syndrome, postvaccinal encephalitis, progressive vaccinia, eczema vaccinatum, generalized vaccinia, and cardiac complications). The risk of developing these adverse events is particularly high in people with immunodeficiency and chronic skin diseases; therefore, these vaccines are contraindicated in PWH and diagnosed with atopic dermatitis [138]. 

Adverse events occurring after vaccination with replication-competent vaccines seem to be closely related to the immune status of the subject. In a study by Tasker et al. of 10 individuals with undiagnosed HIV-1 infection and CD4 counts >200 cells/mm^3^ at the time of smallpox vaccination, none developed adverse events [139]. On the other hand, in a case report by Redfield et al., the patient who developed disseminated vaccinia had a CD4 cells count below 25 cells/mm^3^ and active cryptococcal meningitis [140]. Therefore, in the interim Guidance for Prevention and Treatment of Monkeypox in Persons with HIV Infection, O’Shea et al. do not recommend using ACAM2000 in PWH for the risk of severe adverse effects [141].

In conclusion, the development of third-generation vaccines such as MVA has made it possible to expand the subjects to whom smallpox and MPX vaccines should be administered, including PWH.

### 2.7. Severe Acute Respiratory Syndrome Coronavirus 2 (SARS-CoV-2)

Severe acute respiratory syndrome coronavirus 2 (SARS-CoV-2) belongs to the coronavirus family and shares 79% sequence with SARS-CoV [142]. The main symptoms of Coronavirus Disease 19 (COVID-19) are fever, cough, and dyspnoea; a low proportion complains of gastrointestinal symptoms, anosmia, dysgeusia, headache, and skin lesions [143,144,145,146,147,148,149]. Most people develop asymptomatic or paucisymptomatic forms of infection [150,151]. However, the disease can evolve into a life-threatening systemic inflammation, respiratory failure, and multiorgan dysfunction [152,153,154,155]. Several studies have been conducted to evaluate if having an HIV infection represents a risk factor for developing severe disease. People with HIV with low CD4 cells count or detectable HIV-RNA seem to have an increased risk of severe COVID-19, while people with an undetectable HIV-RNA and a CD4 count higher than 200 cells/mm^3^ appear to have the same risk as people without HIV [156,157,158]. For these reasons, having HIV was considered among the conditions prioritized for receiving a vaccination and eligible for early antiviral treatment against SARS-CoV-2 [159,160].

In addition, some studies showed that HIV treatments could act against SARS-CoV-2 [161,162]; in particular, treatment with tenofovir disoproxil fumarate (TDF)/emtricitabine (FTC) seems associated with a lower risk of infection and disease progression [163,164]. 

At the end of 2020, the first vaccine against SARS-CoV-2 was already available. Currently, nine vaccines have been approved by the WHO and administered worldwide [165,166,167,168,169,170,171,172,173,174], with many others approved only in one country (e.g., EpiVacCorona fully approved in Turkmenistan) or whose trials are still ongoing [175]. 

Several studies have evaluated the vaccine acceptance, efficacy, and safety in PWH.

*Efficacy*

Only one trial to investigate the efficacy of SARS-CoV-2 vaccination in PWH has been conducted, particularly on the AZD1222/ChAdOx1 vaccine. In the interim analysis, they presented data about 103 PWH (52 received vaccine, 51 received placebo) and 58 PWoH (29 received the vaccine, 29 received placebo). All PWH had an undetectable HIV-RNA and a CD4 cells count above 350 cells/mm^3^. In the interim analysis, the authors compared 54 PWH with 50 PWoH who received two vaccine doses. They found that PWH showed cross-reactive binding antibodies to the beta variant and wild-type Asp614Gly. High responders retained neutralization against beta [176]. In the final analysis, they compared 54 PWH who received two doses with 50 PWoH. They have not found a correlation between the magnitude of anti-spike IgG and CD4 cell count, and there is no difference between the two cohorts [177]. 

More studies have been conducted on mRNA vaccines BNT162b2 and mRNA-1273. Schmidt et al. found a significantly lower level of SARS-CoV-2-specific IgA in PWH than in PWoH, indicating a moderately lower functionality of the humoral vaccine response [178,179]. Likewise, Hefdtal et al. and Xu et al. showed that PWH had a lower level of IgG than PWoH [179,180]. Several studies confirmed these data [178,181]. 

Other studies found a significant difference in neutralizing antibody responses between PWH with a CD4:CD8 ratio < 0.5 or less than 200/250 CD4 cells/mm^3^ [182,183,184,185,186]. On the contrary, Portillo et al. found no evidence of poorer viral neutralization in PWH compared to PWoH [187].

Many of these studies, and we agree with them, concluded that PWH might become a target population for prioritization to receive booster vaccinations.

Regarding Ad26.COV2.S, Khan et al. enrolled 73 PWoH and 26 PWH, and as a comparison group, they included unvaccinated participants (28 PWoH and 34 PWH) with prior documented SARS-CoV-2 infection. They found a similar neutralization response in both groups [188].

About the inactivated vaccine, Coronavac and BBiBP CorV, Netto et al. conducted a cohort study including 215 PWH and 296 PWoH. They found that people with less than 500 CD4/mm^3^ had a lower antibody level than people with more than 500 CD4/mm^3^ [189]. In addition, they found lower S-RBD-IgG antibody seropositivity rates and levels in PWH than in PWoH. Similar results were found by Zeng et al. and Liu et al. [190,191]; however, they found a lower antibody level in people with less than 350 CD4 cells/mm^3^. 

Regarding the other inactivated vaccine, WIBP-CorV, Zou et al. found a delayed and low immunogenicity peak in PWH compared to PWoH; however, no significant difference was found in six-month immunogenicity between the two groups [192].

Finally, Gushchin et al. reported data about the Sputnik vaccine, including 24,423 PWH. Of them, 2543 (10.4%) were fully vaccinated, 17,592 (72.0%) were unvaccinated, and 4288 (17.5%) received only one dose. They found a general vaccination efficiency of 76.3%, while in PWH with more than 350 CD4 cells/mm^3^, it was 79.4%. In addition, vaccination avoided hospitalization in 90.1% of cases and gave protection from moderate or severe disease in 97.1%. For the delta variant, they observed a reduction in action (efficiency 65.3%, avoided hospitalization 75.7%, and protection from moderate/severe disease 93.1%) [193].

*Safety*

Most of the studies present in the literature have not reported notable adverse events. In the AZD1222 trial, the authors have not reported any serious adverse events. At the same time, local and systemic reactions occurred during the first seven days after vaccination. The most common were pain at the injection site (49%), fatigue (47%), headache (47%), and malaise (34%) [176]. However, some studies reported a detectable HIV-RNA in a part of the vaccinated subject in the following months [187]. In the Gianserra et al. study, one patient vaccinated with BNT162b2 developed a reversible sensorineural hearing loss 24 h after boosting [185]. Finally, Chaabouni et al. reported a case of herpetic meningoencephalitis after having received one dose of inactivated vaccine [194]. Atiyat et al. reported a case of a 36-year-old man with HIV that had a Varicella-Zoster Virus (VZV) reactivation two days after he received the second dose of BNT162b2 vaccine. To note, he had a low CD4 cell count (158 cells/mm^3^), and he was not on ART (HIV-RNA 20600 copies/mL) [195].

In conclusion, many studies have been published on SARS-CoV-2 vaccination in PWH. The majority agree that the neutralizing antibodies level was lower in PWH than in PWoH, especially when a low CD4 cell count was present. However, the sample size in many studies is small; for this reason, we believe that other studies are needed, with larger sample sizes, to better understand the different vaccines’ efficacy. Until then, PWH represents a high-risk population, and they should have priority in receiving the boosting doses.

### 2.8. Varicella-Zoster Virus

Varicella-Zoster Virus is one of the known herpes viruses which infect humans. The infection confers life-long immunity; however, the virus remains latent in the ganglia and can reactivate. It can provoke two different clinical diseases. The first infection (Varicella or chickenpox) leads to a vesicular rash that usually spreads to the whole body, particularly affecting the face and trunk. While it is usually a self-limited disease, complications such as soft tissue infection, pneumonia, hepatitis, and encephalitis may affect at-risk individuals (e.g., immunocompromised and pregnant women). Herpes Zoster (HZ) reactivation (shingles) usually involves one or two contiguous dermatomes with a painful vesicular rash; among its complications, postherpetic neuralgia is common in older and immunocompromised patients; eyes, visceral, and neurological involvement are also possible. In pregnant women, HZ may cause foetus injuries [196].

Herpes Zoster has always been common among PWH before the introduction of ART. Ever since, its incidence has been declining [197]; however, PWH are still at higher risk of HZ and its complications compared to the general population [198]. Moreover, in people with VZV or HZ, the treatment must be promptly started to be effective. However, even in the best conditions, it is unlikely to protect from post-herpetic neuralgia and other complications alone [199]. Thus, prevention remains the best chance to reduce the burden of the disease, especially on immunocompromised patients.

Regarding chickenpox prevention, two vaccines are available in Europe: Varivax and Varilrix, both live-attenuated vaccines. After reconstitution, one dose (0.5 mL) of Varivax contains no less than 1350 UFP VZV live-attenuated Oka/Merck strain. After reconstitution, one dose (0.5 mL) of Varilrix contains no less than 10^3.3^ PFU VZV live-attenuated Oka/Merck strain. Guideline recommendations are summarized in Table 6.

Several data regarding the safety and efficacy of these vaccines among the general population are available. On the contrary, few data about using live-attenuated VZV vaccines among PWH can be found in the literature. A systematic review including all published literature up to 2013 conducted by the World Health Organization (WHO) confirmed their efficacy in preventing disease of any severity in immunocompetent individuals. The same review reported a possible benefit for HIV-infected children; nonetheless, more evidence was warranted to clarify its role in this population [200]. While further studies were later published regarding safety and efficacy among children living with HIV [201], there are almost no data on adult PWH. One study conducted to assess the role of attenuated VZV vaccine as a booster to prevent HZ among adult PWH suggests its safety and good tolerability among this population [202]. Nonetheless, given the high risk of a possible fulminant or complicated chickenpox course and relying on safety and efficacy data among children, vaccination is recommended in seronegative individuals with ≥200 CD4+ cells/mm^3^ [102,104]. 

Regarding HZ reactivation, live-attenuated VZV vaccines have been used as boosters in children and adults living with HIV [201,203]. Weinberg et al. enrolled 82 subjects with positive serology for VZV; the first group included PWH on stable ART and with >400 CD4+ cells/mm^3^, and the second included PWH with CD4 cells count between 201 cells/mm^3^ and 400 cells/mm^3^ at study entry, and one cohort of HIV-negative subjects. In each cohort, subjects were randomized to Varivax or placebo: the study reported excellent tolerability and moderate immunogenicity in PWH enrolled.

In 2006, the first vaccine against HZ was licensed (Zostavax)—a live-attenuated vaccine. After reconstitution, one dose (0.65 mL) contains no less than 19,400 PFU VZV live-attenuated Oka/Merck strain. Due to its characteristic, few studies are available among immunocompromised patients, including PWH. Therefore, a randomized, double-blind, placebo-controlled, multicenter study has been conducted to assess the efficacy and safety of the heat-attenuated formulation among PWH with less than 200 CD4+ cells/mm^3^ within 90 days before the first dose administration; four doses were administered approximately 30 days apart. Although the vaccine was safe and well-tolerated, results were unsatisfactory regarding immunogenicity, with a weak immunogenic response unlikely to be protective against VZV reactivation [204]. More encouraging results were reported from PWH with a better immunologic profile. A randomized, double-blind, placebo-controlled trial in virally suppressed PWH, and with at least 200 CD4+ cells/mm^3,^ demonstrated the safety and immunogenicity of HZ live-attenuated vaccine even though the 24-weeks follow-up could not guarantee the durability of this effect [205]. However, being a live-attenuated vaccine, some concerns exist about its use in an immunocompromised population.

The recently approved recombinant, adjuvanted HZ vaccine (Shingrix) may be a more suitable alternative. The adjuvanted VZV glycoprotein E subunit vaccine was recently authorized for immunocompromised adults [102,104]. Results from older adults demonstrated efficacy and tolerability [206,207,208]. A phase 1/2a, randomized, observer-masked, placebo-controlled, multicenter trial conducted among PWH confirmed Shingrix’s effectiveness in soliciting an immune response. A two-doses course was associated with a significant increase in cellular and humoral immunity compared with a single dose, while a third dose was reported as not significantly beneficial [209]. The same study reported excellent tolerability, with pain at the injection site and fatigue as the most reported symptoms; no vaccine-related severe adverse event leading to withdrawal was reported [209].

Safety and tolerability among immunocompromised individuals were confirmed by a review of six trials addressing this wide population. Data from oncologic, transplanted, and seropositive patients were reported, confirming the clinically acceptable profile of the vaccine [210]. In addition, real-life data from the Medicare population (n = 15.589.546) confirmed the beneficial impact of Shingrix among immunocompromised individuals. Izurieta et al. reported significantly higher immunogenicity following the second dose, despite lower effectiveness than reported among the general population (64.1% vs. 70.9%) [211]. However, the authors did not provide which were the underlying conditions of the subjects involved. In addition, the study was published only two years after licensure; thus, further surveillance data should follow to confirm preliminary results. 

In conclusion, we have different strategies to address Varicella and Zoster reactivation among a fragile population such as PWH. The newest vaccine (Shingrix) seems promising, effective, and well-tolerated. However, long-term, real-life data are needed to better inform vaccination campaigns.

### 2.9. Neisseria Meningitidis

*Neisseria meningitidis* is a gram-negative, facultatively anaerobic diplococcus that exclusively infects humans. It was first isolated in 1887 from observing the cerebral spinal fluid of patients with meningitis [212].

It is the etiologic agent of severe meningitis and systemic infections that primarily affect children and young adults, named invasive meningococcal disease (IMD). Five main serogroups cause IMD: A, B, C, W, and Y. According to the last ECDC report, updated in 2018, the notification rate remained relatively stable in the last three years. Serogroup B is confirmed to be responsible for most cases (51%), followed by serogroups W and C; Serogroup A is more common in Africa, Asia, South America, and ex-Soviet Republics; Serogroup Y accounts for about one-third of cases in the United States [213]. 

Fever, myalgias, nausea, vomiting, and headache characterize the initial symptomatology of IMD. Subsequently, loss of consciousness, confusion, meningism, and hemorrhagic rash may onset. Treatment should not be delayed more than one hour to reduce the mortality risk; thus, a prompt diagnosis is crucial [214,215].

People with HIV are at increased risk of developing invasive *N. meningitidis* disease, regardless of sexual habits [216]. Some factors appear to be associated with an increased risk of IMD in PWH. Among these, the higher risk of bacteremia compared to patients without HIV infection is relevant [217]. The mortality rate seems directly proportional to the CD4 cell count, suggesting that the elevated risk for IMD among PWH is at least partially a result of HIV-related immune suppression [216]. In addition, atypical infections due to N. meningitidis, such as septic arthritis, have been described in PWH [217,218].

Three types of vaccine are available: the tetravalent MenACWY, against the serogroups A, C, W, and Y; the monovalent vaccine against B serogroup (MenB); the monovalent glycoconjugate-vaccine against meningococcal C vaccine (Menjugate) [219].

The tetravalent vaccines are as follows: (i) MenACWY-D (Menactra), that it is a conjugate vaccine with polysaccharide diphtheria [220]; (ii) MenACWY-CRM (Menveo), a conjugate vaccine with the oligosaccharide diphtheria CRM_197_ [221]; (iii) MenACWY-TT (MenQuadfi), a conjugate vaccine with polysaccharide tetanus toxoid [222].

The available vaccines against serogroup B are as follows: (i) MenB-FHbp (Trumenba) consists of two purified recombinant lipidated FHbp antigens, one from each FHbp subfamily (A and B) [223]; (ii) MenB-4C consists of three recombinant proteins (neisserial adhesin A [NadA], factor H binding protein [FHbp] fusion protein from subfamily B, and neisserial heparin-binding antigen [NhbA] fusion protein), and outer membrane vesicles (OMVs) containing outer membrane protein porin A (PorA) serosubtype P1.4 [224].

All guidelines suggest vaccination for *N. meningitidis* in PWH; however, each guideline suggests a different approach. For example, EACS suggests performing the quadrivalent vaccination every five years according to the risk factors and does not recommend the MenB vaccine [24]. NIH suggests vaccinating all PWH over 18 years old with the quadrivalent vaccine, while MenB is not routinely indicated [23] (Table 7). 

However, few studies have been conducted among PWH to assess the immunogenicity in this specific population. Siberry et al. conducted a Phase I/II trial among children and youth with HIV (11–24 years old). One dose of quadrivalent Polysaccharide Diphtheria Toxoid Conjugate Vaccine was administered to all participants (317). Then, all people with CD4 cells < 15% received a second dose at 24 weeks; the other participants were randomized to receive or not a second dose. They found that immunogenicity was weaker than in the general population, especially for people with low CD4 cells count and detectable viral load [225]. These data were confirmed by Lujan-Zilbermann et al. [226]. Frota et al., in their study among children and young PWH, found that one dose of the quadrivalent vaccine was insufficient and suggested performing the second dose [227,228]. These studies also showed the excellent safety of the vaccines with a very low incidence of adverse events [225,226,227,228].

Regarding the MenB, no studies have been conducted on PWH to assess immunogenicity and safety. Of interest, in a recent study, Raccagni et al. evaluated the incidence of *Neisseria gonorrhoea* in MSM living with HIV with a recent history of sexually transmitted infections [229]. They observed how, during the follow-up (median 3.8 years), people who received two doses of MenB vaccination had a 44% reduced risk of gonorrhoea, confirming what was described by Paynter et al. and Petousis-Harris in the general population [230,231].

Further studies are needed to assess these vaccines’ efficacy in adult PWH. However, due to its high efficacy, vaccination should be recommended for all PWH, especially those with an increased risk of infection [232,233]. A recent review conducted in the United States shows a relatively low vaccination rate, even in newly diagnosed patients [234]. In this regard, we believe that informing people about the cross-efficacy of the vaccine for gonorrhoea could be an incentive for vaccination and an opportunity for counselling about meningitis and sexually transmitted infections [235].

### 2.10. Pertussis, Diphtheria, and Tetanus

Pertussis (Whooping cough) is a highly contagious bacterial infection caused by *Bordetella pertussis* and mainly affects the high respiratory tract [236]. It is effectively preventable by inactivated vaccine, although it does not confer life-long immunity. Whooping cough is considered a childhood disease; however, its prevalence among adults may be underreported. Studies among adult PWH are anecdotal, but a cross-sectional seroprevalence study conducted in the United States showed a 1000-fold higher prevalence among PWH than the general population [237]. In addition, case reports show a severe course of the diseases among people with AIDS [238,239].

Diphtheria is caused by *Corynebacterium diphtheriae* and *Corynebacterium ulcerans*; it affects the high respiratory tract and skin [240]. It is preventable thanks to the toxoid vaccine. Data regarding disease prevalence and severity among PWH are lacking, as are vaccine safety and efficacy among this population; however, it seems that PWH, especially with low CD4 cell count, develop a lower antibody titre than the general population [241,242].

Tetanus is caused by neurotoxins released by *Clostridium tetani*. It causes general rigidity and spasms, leading to respiratory and cardiac failure and death [243]. It is preventable through a toxoid vaccine, with a long, although not lifelong, lasting immunity; reinforcing doses are recommended every 10 years. It is not known if the clinical course and mortality rate differ in PWH compared to the general population; however, data regarding immunogenicity suggest a poorer immunological response in PWH. A trial published in 2019 reported a lower memory response in children with HIV not on ART, while a similar kinetic was reported in children with HIV on ART and children unexposed to HIV [244]. In addition, there was a greater waning in immunity; thus, earlier booster doses should be considered in children with HIV [245]. A study conducted in Senegal confirmed a lower immunogenic response in PWH than in the general population [246]. Dauby et al. estimated the durability of tetanus toxoid-specific seroprotection, finding a half-life of 9.9 years. In addition, in their analysis, people born outside Europe had a shorter half-life (4.4 years), probably due to their low CD4 cell count at the time of immunization and the low CD4 nadir. They concluded that longer intervals of booster vaccination, as recommended in the general population, might not be appropriate in this subgroup of PWH [247]. Regarding safety, no data regarding increased adverse events are reported. 

Pertussis, diphtheria, and tetanus vaccines are often administered together in a pediatric (DTPa) or adult (≥7 years old) formulation with a reduced component of diphtheria and pertussis (dTpa). They can also be found combined with the polio vaccine in a tetravalent formulation (dTpaIPV).

In conclusion, all guidelines reported that the indication for these three pathogens is no different in people with HIV and suggested following standard recommendations.

### 2.11. Streptococcus Pneumoniae

*Streptococcus pneumoniae* is a Gram-positive bacteria with alfa- and beta-hemolytic features in aerobiotic and anaerobiotic conditions. It causes a broad spectrum of infections, such as pneumonia, meningitis, otitis, bronchitis, conjunctivitis, sepsis, osteomyelitis, and others [248,249]. 

HIV-related immunological deficiency exposes PWH to an increased risk of pneumococcal infections and severe manifestations [250,251]. The risk of pneumococcal infection is from 10- to 100-fold higher in PWH receiving and not receiving ART; the risk increases with a lower CD4 cell count (<350 cells/mm^3^) [250,252].

To date, four different vaccines are available: 23-valent unconjugated purified polysaccharide vaccine (PPSV23), 13-valent vaccine conjugate vaccine (PCV-13), 15-valent pneumococcal conjugate vaccine (PCV15), and the 20-valent pneumococcal conjugate vaccine (PCV20). The first conjugate vaccine, PCV-7, is no more used (Table 8). 

Several studies about the pneumococcal vaccine in PWH have been conducted with variable results.

The recommendation for the general population is to utilize either PCV20 alone or PCV15 in association with the PPSV23 [253]. However, HIV guidelines have different suggestions due to the lack of data in PWH. For example, EACS suggest administering one dose of any PCV vaccine [13,15,20] to all PWH according to national guidelines, even if they already received PPSV-23 [24]. In addition, they recommend one dose of PPSV-23 in those who received PCV-13 or PCV-15. NIH, on the contrary, suggests PPSV-23 in all people with more than 200 CD4/mm^3^. In those who received PCV-13, NIH suggests administering one dose of PPSV-23 [23] (Table 9).

It was suggested that PPSV-23 might not be the appropriate vaccination strategy for PWH because of some characteristics of this vaccine. Specifically, it contains purified polysaccharide antigens of the pneumococcal capsule and produces immunity through activating B-cells without T-cells involvement. Because of this, no immune memory is produced, and adjunctive doses do not elicit an additional immune response [254]. On the other hand, conjugated vaccines like PCV stimulate B- and T-cell activation, providing a sustained immune memory.

Lesprit et al. enrolled 213 adults infected with HIV and randomized them to receive either one dose of PCV-7 followed by one dose of PPSV-23 after four weeks or one dose of PPSV-23 at week four. The two-dose group showed higher immune responses than those receiving only one PPV dose at weeks 8 and 24 [255]. Similar results were obtained by Feikin et al. in a randomized trial where they found a better immune response in those who received two doses of PCV-7 or one of PCV-7 and one of PPSV-23 versus a third group that received one dose of placebo and one of PPSV-23 [256]. In addition, Bhorat et al. conducted a trial on PWH by administering three doses of PCV-13 followed by one dose of PPSV-23 at 1-month intervals with good tolerability and showing that PWH achieved a significant immune response after the first dose of PCV-13, with only modest increases in antibody titres following the other doses.

The only clinical efficacy study was performed by French et al. from 2003 to 2007, involving both people with CD4 cell count lower and higher than 200 cells/mm^3^; among the 439 PWH enrolled in the study, only 13% were receiving ART. Nevertheless, they found that in the group vaccinated with PCV-7, the one-year efficacy was 85% [257]. A meta-analysis by Garrido et al. conducted in 2020 concluded that the combination of PCV-13 and PPSV-23 has good immunogenicity; however, the durability of this vaccination remains unknown. In addition, data suggested delaying the PCV administration until the CD4 cells count is above 200 cells/mm^3^.

In 2022, Mohapi et al. published a randomized, double-blinded clinical trial to compare the immunogenicity and safety of PCV-15 and PCV-13; they included 302 PWH. PCV-15 was generally well tolerated; immune responses were elicited for all 15 pneumococcal serotypes. However, no clinical data about this vaccine are available.

In conclusion, there is scarce clinical evidence about the efficacy of PCV in PWH. Further studies are needed both for clinical efficacy and to assess the immunogenicity of the new PCV vaccine in this specific population. However, considering the severity of pneumococcal pneumonia among immunocompromised individuals, physicians should recommend the combination of PCV-PPSV, according to international guidelines. Special attention should be given to PWH with <200 CD4 cells/mm^3^.

### 2.12. Vaccines for Travel

The immunization of international travelers is mandatory to prevent the spread of infections between countries and reduce the risk of severe disease and death [258]. However, these vaccines’ acceptance is low [259,260,261,262]. Below, we briefly report the suggested vaccination for travelers and the available information and recommendation for PWH.

- **Cholera**: it could be present in the area without a clean water supply or modern sewage system or in case of environmental changes due to natural disasters (such as tsunamis) or human-driven events (such as wars or massive migrations). People with HIV have a higher risk of contracting the infection and suffering severe consequences [263]. Many vaccines are available for cholera. CVD103-HgR (replicating—live-attenuated) has been proven safe and immunogenetic in PWH; however, it is not currently available worldwide [264]. According to the NIH, it could be administered in at-risk PWH with more than 200 CD4 cells/mm^3^. If CVD103-HgR is unavailable, according to BHIVA guidelines, WC/rBS vaccine (inactivated) could be administered. It is essential to know that PWH with less than 100 CD4/mm^3^ may be expected to respond poorly to it [265].
- **Flu Vaccine**: see paragraph “Influenza”.
- **HAV**: see paragraph “HAV”.
- **HBV**: see paragraph “HBV”.
- **Japanese encephalitis (JEV)**: it is caused by a *Flavivirus* transmitted by mosquito bites. This virus is present only in Asia. In most people, it is asymptomatic; however, it is symptomatic in approximately 1 patient out of 250 infections. The severity of infection ranges from flu-like to encephalitis, which could be life-threatening in 20–30% of cases. Many vaccines for JEV are available worldwide (inactivated, live-attenuated, and live-recombinant) [266]. For PWH, the inactivated vaccine (IXARO^®^) is suggested. However, no study in adult PWH have been conducted, and only BHIVA guidelines provide recommendations regarding this vaccine [25].
- ***N. meningitidis***: see paragraph “*Neisseria meningitidis*”.
- **Rabies**: it is transmitted by infected animals (present worldwide), generally with a bite or a scratch. It causes acute encephalomyelitis, sometimes associated with ascending flaccid paralysis. If not rapidly treated with immunoglobulins, patient death is very likely, with only a few reported survivors [240].
  The vaccine against rabies is inactivated and administered in three doses. The vaccine induces satisfactory antibody production in more than 95% of vaccinated subjects. However, in PWH, the immunogenicity is influenced by CD4 count and viral load; in particular, low or absent antibody responses were reported in some patients with CD4 cell counts <250 CD4/mm^3^ [267,268].
- **Polioviruses**: three serotypes of polioviruses could infect humans. They spread through the fecal–oral and respiratory routes. They usually cause gastrointestinal symptoms, but in some cases, they could give severe neurological manifestations, including meningitis, encephalitis, and poliomyelitis syndrome with acute onset of flaccid paralysis. Two vaccines against polioviruses are available [269]; however, the live-attenuated oral poliovirus vaccine is not used anymore in many countries due to its side effects since it could cause vaccine-associated paralytic polio, especially in immunocompromised people, including PWH [270]. The trivalent inactivated poliovirus vaccine is the most used, combined with tetanus and the diphtheria toxoid (Td/IPV). People with HIV should receive three doses if they are not vaccinated or have an uncertain vaccination history, followed by two booster doses after 5 and 10 years. Then, a booster every 10 years is suggested.
- **Tuberculosis:** it is present worldwide, with a higher incidence in Asia, Africa, and South America. In most people, it remains latent; however, latent TB could reactivate in 5–15% of immunocompetent adults [271,272]. In PWH, the risk of activation is higher, especially in those people with a low CD4 cells count. The Bacille Calmette-Guerin (BCF) vaccine is a live-attenuated vaccine from *Mycobacterium bovis* strains. Its efficacy is controversial since the protection rate varies widely among different trials [273]. According to HIV guidelines, the BCG vaccine is contraindicated in PWH regardless of CD4 cells count, ART, viral load, and clinical status since some studies described a higher risk of local and systemic complications among this population, including disseminated BCG [21,25].
- **Typhoid Fever**: it is a cosmopolitan infection; however, higher-risk areas are characterized by poor sanitation and hygiene (Africa, India, South-East Asia, and South America). Three vaccines are available: (i) Vi (polysaccharide vaccine—one intramuscular dose); (ii) Ty21a (live-attenuated—tablets); (iii) Combined with HAV (polysaccharide vaccine—intramuscular). Ty21a is not recommended in PWH, especially in those with less than 200 CD4 cells/mm^3^, since it contains live samples of *Salmonella typhi*.
  On the contrary, VI vaccination is recommended in all PWH and should be performed at least one week before exposure [274].
- **Yellow fever:** it is caused by a virus transmitted through the bite of an infected *Aedes aegypti* mosquito. It is present in tropical and subtropical regions of Africa and South America. The infection is often symptomatic [275]; however, it sometimes causes severe hepatitis, jaundice, and bleeding, with high mortality rates. It is not known if PWH have an increased risk of severe forms. The vaccine contains a replicating live-attenuated virus. A single dose protects around 90% after 10 days and 99% after 30 days [276]. In 2014 a systematic review investigated the immunogenicity and safety of the yellow fever vaccine in PWH. They found that PWH (compared with PWoH) developed significantly lower concentrations of neutralizing antibodies in the first year post-immunization; however, the decay patterns were similar for recipients regardless of HIV infection. Furthermore, no study patient with HIV infection suffered serious adverse events due to vaccination [277]. However, since it contains a replicating live-attenuated virus, this vaccine is suggested only in PWH with more than 200 CD4/mm^3^ and aged <60 years old.

In conclusion, many vaccines are not recommended in PWH with low CD4 counts. A thorough analysis of the pros and cons is needed for these patients. In addition, we recommend postponing travels, when possible, until immunological recovery.

## 3. Conclusions

Vaccinations represent a powerful tool to avoid vaccine-preventable infectious diseases also in PWH. Therefore, clinicians should carefully collect the history of vaccinations in all new patients and regularly check the presence of antibodies for each vaccine-preventable pathogen, especially in people with low CD4 numbers, who have a higher antibody-waning rate. Although vaccinations for PWH are strongly recommended, data on immunogenicity, tolerability, and clinical efficacy are limited for this specific population. For this reason, further studies are needed to assess these features and harmonize different guidelines. In addition, the acceptance rate can and must be improved.

## Figures and Tables

**Table 1:** Comparison of five HIV guideline recommendations for the HAV vaccine administration.

	BHIVA [22,25]	EACS [24]	NIH [23]	SIMIT [27]	WHO [26]
Who to vaccine?	According to risk profile (travel, close contact with children, MSM, IVDU, active hepatitis B or C infection, chronic liver disease), and with a negative anti-HAV IgG antibodies	According to risk profile (travel, close contact with children, MSM, IVDU, active hepatitis B or C infection, chronic liver disease), and with a negative anti-HAV IgG antibodies	Any person without evidence of immunity to HAV	According to risk profile (travel, close contact with children, MSM, IVDU, active hepatitis B or C infection, chronic liver disease), and with a negative anti-HAV IgG antibodies	No specific recommendation
Difference for people with low CD4/mm^3^	>350 CD4/mm^3^: two vaccines doses at 0 and 6 months <350 CD4/mm^3^: three vaccines doses at 0, 1, and 6 months	No	<200 CD4 with risk factors: do vaccination and check antibodies response after 1–2 months. If negative, revaccinate when CD4 are >200. <200 CD4/mm^3^ without risk factors: waiting for CD4 > 200/mm^3^	No	No specific recommendation
Boosting?	Every 10 years	NP	NP	The cited BHIVA’s recommendation of performing a booster every 10 years in high-risk people	NP

NP: not present.

**Table 2:** Comparison of five HIV guideline recommendations for the HBV vaccine administration.

	BHIVA [22,25]	EACS [24]	NIH [23]	SIMIT [27]	WHO [26]
Who to vaccinate?	All if seronegative	All if seronegative	All if seronegative	All if seronegative	All if seronegative
Type of vaccine and doses	Yeast-based: 40 µg Adjuvanted: 20 µg Four doses: 0, 1, 2, 6 months	According to national guidelines	Yeast-based: 40µg Adjuvanted: 20 µg Three doses	Yeast-based: 40 µg Adjuvanted (preferred): 20 µg Three doses	Suggest using double doses
Target IgG	>100 UI/L 8 weeks after the last doses	>100 UI/L	≥10 mIU/mL 8 weeks after the last doses	>100 UI/L	>100 UI/L
Occult HBV*	One dose; check HBsAb two weeks later; if HBsAg < 10 IU/L, offer full vaccination	NP	NP	One dose; check HBsAb two weeks later; if HBsAg < 10 IU/L, offer full vaccination	NP
Differences for people with low CD4/mm^3^	No differences in doses; repeat HBsAb screening more frequently if CD4 cell/mm^3^ < 350	For people with “particularly low CD4”, consider a double dose (40 µg) or use a more immunogenetic vaccine	No difference in doses. For non-responder people with CD4/mm^3^ < 200: delay re-vaccination until CD4 > 200/mm^3^	No difference in doses. For non-responder people with CD4/mm^3^ < 500: delay re-vaccination until CD4 > 500/mm^3^	NP
Boosting	People with HBsAg < 10 UI/L: three more doses People with HBsAg < 100 UI/L but >10 UI/L: one dose	People with HBsAg < 10 UI/L: three more doses People with HBsAg < 100 UI/L but >10 UI/L: one dose	Non-responder: revaccinate with 3–4 doses For people whose HBsAg level fall below 10 UI/L: one dose if not receiving tenofovir-based regimen	People with HBsAg < 10 UI/L: three more doses People with HBsAg < 100 UI/L but >10 UI/L: one dose	People with HBsAg < 10 UI/L: three more doses People with HBsAg < 100 UI/L but >10 UI/L: one dose

* HBsAg negative, HBcAb positive, and HBsAb negative. NP: not present.

**Table 3:** Randomized controlled trials on HBV vaccination among PWH who never received vaccine.

Study	Year	Included Patients	Schedule	Rates of Response	*p*-Value
Fonseca et al. [61]	2005	210	20 µg vs. 40 µg Month 0–1–6	34% vs. 47%	0.07
Cornejo-Suarez et al. [60]	2006	79	10 µg vs. 40 µg Month 0–1–6	60% vs. 61.5%	-
Launay et al. [62]	2011	437	20 µg Month 0–1–6 40 µg Month 0–1–2–6 4 mg ID Month 0–1–2–6	65% 82% 77%	- <0.01 0.02
Chaiklang et al. [64]	2013	132	20 µg Month 0–1–6 40 µg Month 0–1–2–6 4 mg ID Month 0–1–2–6	70.4% 86.4% 88.6%	- 0.119 0.062

**Table 4:** Randomized controlled trials on HBV vaccination among PWH who did not respond to vaccine.

Study	Year	Included Patients	Revaccination Schedule	Rates of Response	*p*-Value
Psevdos et al. [67]	2010	101	40 µg (3 doses) vs. 20 µg (3 to 8 doses) after classic schedule	85% vs. 59%	0.006
Rey et al. [68]	2015	178	Double vs. standard schedule	54% vs. 31%	0.001
Vargas et al. [69]	2021	107	20 µg vs. 40 µg Month 0-1-2	80% vs. 39.1%	0.01

**Table 5:** Comparison of five HIV guideline recommendations for HPV vaccine administration.

	BHIVA [22,25]	EACS [24]	NIH [23]	SIMIT [27]	WHO [26]
Who to vaccinate?	All aged ≤ 26 yo; MSM and women aged < 40; Defer if CD4 < 200/mm^3^	All people aged between 9 and 45	All aged ≤ 26 For people between 27 and 45 years old, depending on risk factors	All aged ≤ 26 For people with more than 26 years evaluate risk/benefit	Girls aged between 9 and 14; females aged ≥ 15 years or males are recommended only if this is feasible, affordable, cost-effective, and does not divert resources from vaccination of the primary target population
Type of vaccine and doses	If available, prefer the 9-valent vaccine; otherwise, use the 4-valent vaccine For both, perform three doses: 0, 1–2, and 6 months	Prefer the 9-valent vaccine	If available, prefer the 9-valent vaccine; otherwise, use the 4-valent vaccine For both, perform three doses: 0, 1–2, and 6 months	If available, prefer the 9-valent vaccine; otherwise, use the 4-valent vaccine For both, perform three doses	Depending on which is available Performing three doses
Differences for people with low CD4/mm^3^	Naïve people with CD4 < 200/mm^3^: deferred until the ART starts	NP	NP	NP	NP
People with HPV disease	Perform vaccine despite age to reduce risk of recurrences	Perform vaccine despite age to reduce risk of recurrences	NP	NP	NP

NP: not present.

**Table 6:** Comparison of five HIV guideline recommendations for the Varicella-Zoster vaccine administration.

	BHIVA [22,25]	EACS [24]	NIH [23]	SIMIT [27]	WHO [26]
**VZV**					
Who to vaccinate?	All with a negative or uncertain history of chickenpox or shingles	All with a negative serology	All with a negative serology	All with a negative serology	All if seronegative
IgG testing	Yes, to determine susceptibility to primary infection and reactivation	Yes	Only HIV without a history of prior varicella or varicella vaccination	NP	NP
Differences for people with low CD4/mm^3^	Only in people with CD4 > 200/mm^3^	Contraindicated if CD4 count <200 cells/μL (14%) and/or AIDS	Only in people with CD4 > 200/mm^3^	Only in people with CD4 > 200/mm^3^	NP
**Zoster prevention**					
Who to vaccinate?	VZV IgG seropositive with more than 60 years	NP	VZV IgG seropositive with more than 18 years	NA	NP
Differences for people with low CD4/mm^3^	Only in people with CD4 > 200/mm^3^	NP	No data identify the optimal vaccination timing for persons with a CD4 < 200/mm^3^. Some experts would administer the RZV vaccination series after CD4 count recovery	NA	NP

NA: not available at the moment of guidelines publication. NP: not present.

**Table 7:** Comparison of five HIV guideline recommendations for the Neisseria meningitidis vaccine administration.

	BHIVA [22,25]	EACS [24]	NIH [23]	SIMIT [27]	WHO [26]
Who to vaccinate?	All aged < 25 if not already vaccinated or if they received the last MenC doses below the age of 10 years; Presence of specific risk factors: asplenia or persistent complement component deficiency; Risk of exposure through travel	According to the risk profile: - Travel - MSM - Contact with Children	All aged > 18 years if not already vaccinated	All people	Recommended for children in some high-risk populations
Type of vaccine	MenC, MenACWY, Men B in people < 25 years MenC, MenB, and/or MenACWY for high-risk people; MenACWY: exposed to travel	MenACWY; MenB according to national guidelines	MenACWY; MenB not indicated	MenACWY; MenB	NP
Interval doses	Two doses with 2 months interval	Two doses with 2 months interval	Two doses with 2 months interval	MenACWY: two doses with 2–3 months interval MenB: two doses with at least one-month interval	NP
Booster	MenACWY every five years if ongoing risk through travel or due to underlying condition	NP	Repeat vaccination every 5 years throughout life	Considerer repeat vaccination with MenACWY every 5 years to keep high immunity	NP

NP: not present.

**Table 8:** Comparison between the four available vaccines for *Streptococcus pneumoniae*.

	PPSV-23	PCV-13	PCV-15	PCV-20
Year of introduction	1983	2010	2021	2021
Serotypes included	1,2, 3, 4, 5, 6B, 7F, 8, 9N, 9V, 10A, 11A, 12F, 14, 15B, 17F, 18C, 19F, 19A, 20, 22F, 23F, 33F	1, 3, 4, 5, 6A, 6B, 7F, 9V, 14, 18C, 19F, 19A, 23F	1, 3, 4, 5, 6A, 6B, 7F, 9V, 14, 18C, 19A, 19F, 22F, 23F, 33F	1, 3, 4, 5, 6A, 6B, 7F, 8, 9V, 10A, 11A, 12F, 14, 15B, 18C, 19F, 19A, 22F, 23F, 33F
Advantages	Low Cost; High number of serotypes; Trial conducted in PWH	Longer lasting immunity; Trial conducted in PWH	Longer lasting immunity; High number of serotypes; Trial conducted in PWH only for immunogenicity	High number of serotypes
Disadvantages	Need of re-vaccinations every 3–5 years	Few serotypes covered	No clinical data on the efficacy	No clinical data about efficacy; Lack of immunogenicity in immunocompromised hosts

**Table 9:** Comparison of five HIV guideline recommendations for the *Streptococcus pneumoniae* vaccine administration.

	BHIVA [22,25]	EACS [24]	NIH [23]	SIMIT [27]	WHO [26]
PCV-13	All once (PVC-13)	If PCV-15 is not available	Non more recommended	All once	NP
PPV-23	At-risk individuals, according to national plan	At-risk individuals, according to national plan: one dose of PPSV-23 after PCV-13 or 15	Only in people previously vaccinated with PCV-13; Booster dose after five years in those previously vaccinated with PCV-13 or PPV-23; If the second PPV-23 dose is performed before 65 years, a third dose with 5 years interval should be offered	One dose in those previously vaccinated with PCV-13 with 2 months interval; Two doses in those never vaccinated with one-year intervals, and a third dose after five years	NP
PCV-15	NA	All once	All once (except those vaccinated with PCV-20), including those already vaccinated with one PPV-23 dose	NA	NP
PCV-20	NA	All once if not vaccinated before	All once (except those vaccinated with PCV-15), including those already vaccinated with one PPV-23 dose	NA	NP

NA: not available at the moment of guidelines publication. NP: not present.

## Data Availability

Not applicable.
